# Plant-Based Decellularization: A Novel Approach for Perfusion-Compatible Tissue Engineering Structures

**DOI:** 10.4014/jmb.2401.01024

**Published:** 2024-02-29

**Authors:** Md Mehedee Hasan, Ashikur Rahman Swapon, Tazrin Islam Dipti, Yeong-Jin Choi, Hee-Gyeong Yi

**Affiliations:** 1 Department of Convergence Biosystems Engineering, College of Agriculture and Life Sciences (CALS), Chonnam National University, Gwangju 61186, Republic of Korea; 2 Interdisciplinary Program in IT-Bio Convergence System, Chonnam National University, Republic of Korea; 3 Department of Advanced Biomaterials Research, Korea Institute of Materials Science (KIMS), Changwon 51508, Republic of Korea

**Keywords:** Green tissue engineering, plant scaffolds, vascularized structures, regenerative innovation, sustainable bioengineering

## Abstract

This study explores the potential of plant-based decellularization in regenerative medicine, a pivotal development in tissue engineering focusing on scaffold development, modification, and vascularization. Plant decellularization involves removing cellular components from plant structures, offering an eco-friendly and cost-effective alternative to traditional scaffold materials. The use of plant-derived polymers is critical, presenting both benefits and challenges, notably in mechanical properties. Integration of plant vascular networks represents a significant bioengineering breakthrough, aligning with natural design principles. The paper provides an in-depth analysis of development protocols, scaffold fabrication considerations, and illustrative case studies showcasing plant-based decellularization applications. This technique is transformative, offering sustainable scaffold design solutions with readily available plant materials capable of forming perfusable structures. Ongoing research aims to refine protocols, assess long-term implications, and adapt the process for clinical use, indicating a path toward widespread adoption. Plant-based decellularization holds promise for regenerative medicine, bridging biological sciences with engineering through eco-friendly approaches. Future perspectives include protocol optimization, understanding long-term impacts, clinical scalability, addressing mechanical limitations, fostering collaboration, exploring new research areas, and enhancing education. Collectively, these efforts envision a regenerative future where nature and scientific innovation converge to create sustainable solutions, offering hope for generations to come.

## Introduction

Tissue engineering has emerged as a groundbreaking field that seamlessly integrates materials science, mechanical engineering, clinical medicine, and genetics to revolutionize regenerative medicine [1]. Central to this transformative domain is the intricate interplay of porous three-dimensional (3D) scaffolds meticulously designed to create an optimal environment for tissue and organ regeneration [2]. The selection of scaffold materials has become a critical factor that requires consideration of biocompatibility, biodegradability and mechanical properties. To push the boundaries of conventional tissue engineering, researchers are increasingly exploring innovative solutions. One of these promising avenues involves the incorporation of natural biopolymers [3]. This article explores the burgeoning field of plant-based decellularization, an innovative approach poised to redefine the landscape of scaffold design and tissue engineering [4]. Tissue engineering, at its core, relies on a complex synergy of different scientific disciplines, ranging from materials science to genetics [4]. The aim of this field is to exploit the regenerative potential of living cells to repair or regenerate damaged tissues and organs caused by diseases or trauma. The utilization of 3D scaffolds plays a pivotal role in providing a supportive microenvironment for cellular growth and tissue formation, which is central to tissue engineering [5, 6].

Various of scaffolds made from different materials using different production methods have been used to rebuild a spectrum of tissues and organs within the human body [7]. Critical considerations in scaffold design include biocompatibility, biodegradability, mechanical properties, scaffold architecture, and manufacturing technologies [8]. The ideal scaffold should mimic the extracellular matrix (ECM) of native tissues and provide insights to guide cell behavior and tissue regeneration [9]. Incorporation of natural biopolymers derived from both animal and plant sources has gained prominence due to their potential influence on cell behavior and biocompatibility. However, problems such as poor mechanical properties and rapid biodegradability have prompted researchers to refine these biomaterials. Among the myriad natural sources, plant-based polymers have attracted attention due to their sustainability, abundance, and cost-effectiveness [10]. Plants and plant-derived materials offer a unique opportunity to revolutionize tissue engineering. This article focuses on an innovative approach to plant-based decellularization, a process involving the removal of cellular components from plant structure, leaving behind acellular scaffolds. The advantages of this approach are numerous, starting with the abundance and rapid growth of a large number of plant species, providing a cost-effective and sustainable source of scaffold materials [3].

**Fig. 1:**
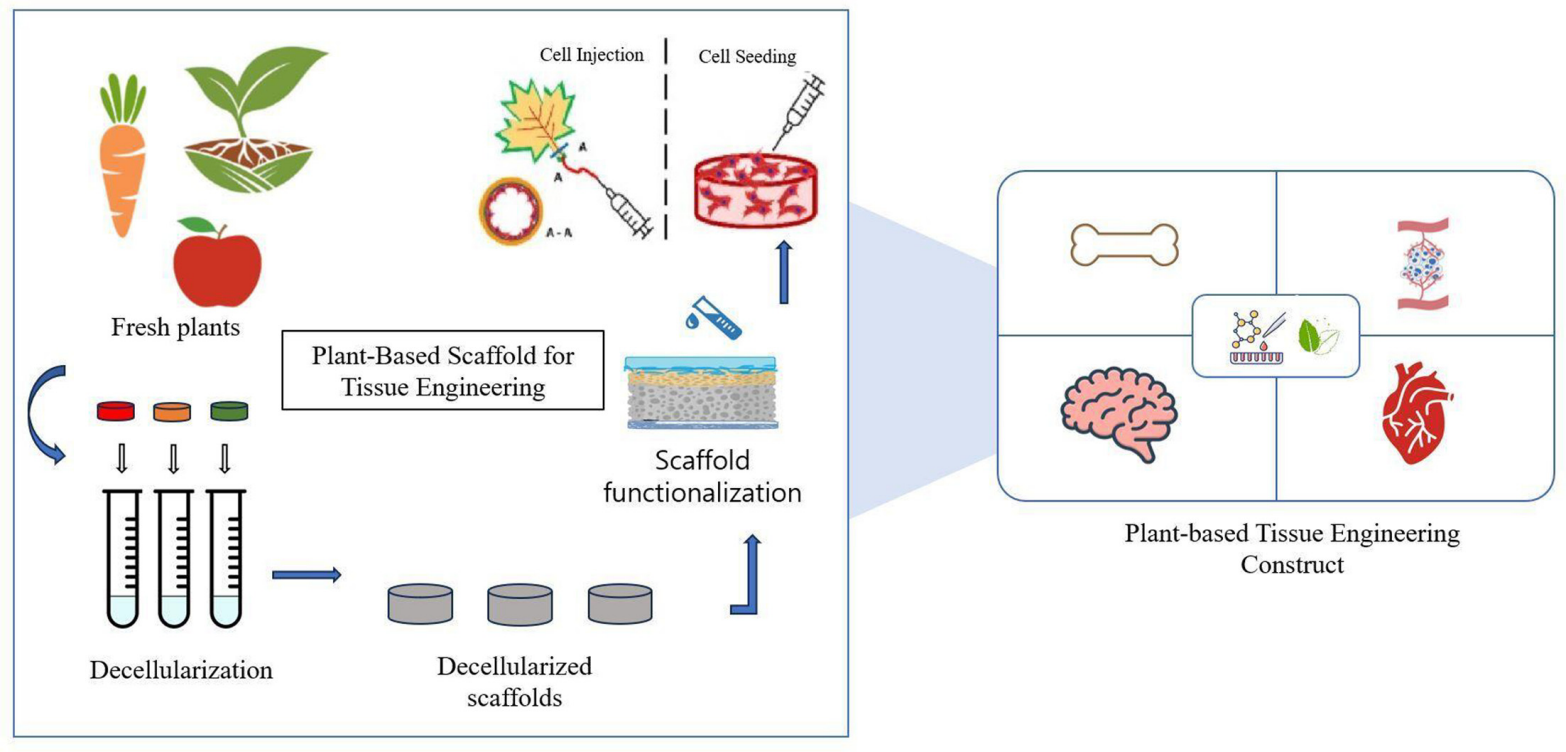
Illustrative depiction of the transformative potential of plant-based decellularization in regenerative medicine and tissue engineering. This schematic encapsulates the intricate process of scaffold development, modification, and vascularization, highlighting the eco-friendly and cost-effective nature of plant-derived materials.

In addition, while the low cost and abundant sources of plant-based materials are highlighted, it's essential to underscore their unique chemical and structural properties that can offer tailored interactions with cells, potentially enhancing scaffold functionality and biocompatibility. Additionally, the renewable nature of plant resources aligns with sustainable practices, making plant-based decellularization an environmentally conscious choice in tissue engineering advancements (Fig. 1).

Natural biopolymers derived from plants, including proteins, carbohydrates, lignin, and extracts, offer a variety of [11] scaffold assembly options [12]. Their biocompatibility, coupled with their ability to shape cell behavior through chemical signals, make them promising candidates for tissue engineering applications [13]. However, their inherent limitations, such as poor mechanical properties and rapid biodegradability, have spurred researchers to explore innovative strategies. Post-spinning cross-linking, a procedure addressing the mechanical limitations by enhancing the structural integrity of natural biopolymers, is a notable approach [14]. In addition, blending natural polymers with synthetic counterparts has shown promise in overcoming these challenges, potentially yielding ideal scaffolds for tissue engineering applications [15]. Synthesizing natural and synthetic elements is crucial for the development of scaffolds that balance biocompatibility, mechanical strength and durability. Traditionally, animal-derived tissues have been the primary source of decellularized scaffolds, but they are limited in terms of cost, availability, and ethics [16]. Plant-based decellularization has emerged as a sustainable and scalable alternative. Furthermore, recent advancements in the development of 3D in vitro biomimetic tissue models using hydrogel-based biomaterials have garnered significant attention in tissue engineering research. Hydrogels, known for their biomimetic properties mirroring the native tissue microenvironment, offer a promising platform for creating intricate tissue structures in vitro . However, challenges persist in achieving optimal mechanical strength solely through natural polymers for scaffold fabrication. Novel scaffold fabrication methods, such as indirect 3D-bioprinting technology utilizing natural polymers like silk fibroin, have emerged to address these limitations. These approaches enable the creation of biocompatible scaffolds with tunable mechanical properties, offering versatility for various tissue engineering applications. As example, the use of plant scaffolds offers significant advantages over existing biomaterials, particularly in addressing vascularization efforts and associated difficulties. Leveraging existing plant vascular systems as blood vessels simplifies the process of vascularization in tissue engineering [11]. This approach streamlines the establishment of functional vasculature in engineered tissues, reducing complexities and enhancing overall feasibility. Thus, incorporating plant scaffolds into tissue engineering strategies represents a promising avenue for overcoming challenges related to vascularization. Decellularization involves the removal of cellular components from a tissue or organ, leaving behind an acellular scaffold composed of ECM [17]. The unique advantage of plant-based decellularization lies in the inherent similarities between plant and animal vascular network structures. According to Murray’s law, plants have a tapered, branching network reminiscent of the human cardiovascular system. Leveraging these similarities offers a novel strategy for creating perfusable scaffolds for tissue engineering [18].

Plant-based decellularization involves the application of decellularization techniques to various plant species and tissues, resulting in the generation of acellular, pre-vascularized tissue engineering scaffolds [19]. In addition to cost-effectiveness and sustainability, creating perfusable structures addresses a critical limitation in current bio-engineering approaches, i.e. the lack of functional vascular networks [20]. Current bioengineering techniques face challenges in the creation of patent perfusion vessels, limiting the size of grafts that can be engineered while maintaining viability. Plant-based decellularization represents a paradigm shift in the use of the intricate vascular networks of plants to create scaffolds that support perfusion [21]. This innovation has a significant promise for the development of tissue engineering solutions in clinical practice. Recognizing the broader implications of this approach is essential for understanding the intricacies of plant-based decellularization [22]. The advantages extend beyond the limitations of current bioengineering techniques. The abundance and rapid growth of plant species is a readily available and cost-effective source of scaffold materials in line with the principles of sustainability and green chemistry [10]. Moreover, while much attention has been given to using the plant itself as a scaffold after decellularization, exploring the properties of plant-based hydrogels and their cross-linking methods could offer valuable insights for future applications. Plant-based hydrogels, with their unique structural characteristics and biocompatibility, hold significant promise in tissue engineering. Understanding the cross-linking mechanisms and manipulation techniques of these hydrogels is crucial for optimizing their mechanical properties and biodegradability in scaffold design. In subsequent sections, we will delve into the properties and potential applications of plant-based hydrogels, shedding light on their role in advancing tissue engineering methodologies.

The field of tissue engineering is at the precipice of a transformative breakthrough in plant-based decellularization [23]. Utilization of plant-derived materials, coupled with innovative decellularization techniques, opens new frontiers in scaffold design [24]. The possibility of creating perfusable structures and overcoming the challenges of vascularization holds promise for advancing tissue engineering solutions. In the following sections, we unravel the intricacies of plant-based decellularization and provide a comprehensive understanding of its potential, challenges and implications for the future of regenerative medicine. The specific objective of this study is to comprehensively explore the potential of plant-based decellularization as a revolutionary approach for generating perfusable tissue engineering structures. In the following sections, we unravel the diverse roles of plant-based polymers, assess the advantages and challenges associated with plant-derived scaffold materials, and discuss the implications of this innovative technique in overcoming current tissue engineering limitations. Additionally, it's imperative to compare and explain the pros and cons of plant-based and existing polymer-based biomaterials in terms of materials. While plant-based materials offer sustainability, unique chemical properties, and potential cost-effectiveness, existing polymer-based biomaterials may offer superior mechanical properties and a longer degradation profile. Understanding these differences is crucial in selecting the most suitable materials for specific tissue engineering applications. Therefore, in subsequent sections, we will delve into a comparative analysis of plant-based and polymer-based biomaterials to provide a comprehensive understanding of their respective advantages and limitations in scaffold design and tissue engineering. Moreover, exploring the effect of certain plants on regenerating specific tissues could provide valuable insights into tissue engineering. Plants possess a rich array of bioactive compounds that may influence tissue regeneration in unique ways. Future research could investigate the specific effects of plant-derived compounds on tissue regeneration and tailor scaffold designs accordingly to enhance tissue-specific regeneration outcomes. So that, future research will explore the potential of plant-based materials in influencing tissue-specific regeneration processes and discuss avenues for further research in this area.

### Development of Plant Decellularization

Plant decellularization has emerged as a transformative and sustainable approach in the dynamic landscape of tissue engineering, promising to redefine conventional scaffold design [25]. This novel technique involves the meticulous removal of cellular components from plant structures, yielding acellular scaffolds rich in ECM composition [9]. Plant-based decellularization is inspired by the traditional methods applied to animal tissues and exploits the distinctive advantages of plants. Scientists have increasingly focused on plant-derived polymers, looking for abundant, sustainable, and cost-effective scaffold materials that include proteins, carbohydrates, lignin, and extracts. However, challenges, such as poor mechanical properties and rapid biodegradability, have led to the investigation of innovative strategies, such as post-spinning cross-linking and blending natural polymers with synthetic polymers. At the core of the transformative potential of plant-based decellularization is its ability to generate acellular, pre-vascularized tissue engineering scaffolds, addressing limitations associated with traditional animal-derived tissues, including cost, availability, and ethical considerations [26]. The inherent similarities between plant and animal vascular network structures provide a unique avenue for creating perfusable scaffolds, crucial for establishing functional vascular networks in tissue engineering [7]. This burgeoning field extends beyond the resolution of the challenges posed by current bioengineering techniques since the abundant and rapid growth of various plant species makes them readily available and cost-effective sources of scaffold materials, which is consistent with sustainability principles [27].

As researchers explore the intricacies of plant-based decellularization, the potential to create perfusable tissue engineering structures is becoming more and more evident, leading to transformative applications in regenerative medicine. Recent studies exploring a spectrum of plant species and tissues and applying decellularization techniques to generate acellular scaffolds highlight the potential of this method in overcoming the challenges associated with current bioengineering approaches [28]. This innovative technique has significant promise for advancing tissue engineering solutions in clinical applications and addressing the growing demand for effective regenerative treatments [9].

Holistic exploration of plant-based decellularization is complemented by numerous studies that provide insights into the potential of plant-based polymers for tissue-engineering applications, comprehensive analysis, and understanding [21]. These references highlight the depth and breadth of plant-based decellularization research. The development of plant decellularization is a testament to the ongoing evolution of tissue engineering, with researchers embracing the unique attributes of plant-derived materials and innovative decellularization techniques to open new frontiers in scaffold design [29]. The potential to create perfusable structures, together with addressing the challenges of vascularization, holds great promise for the development of novel tissue engineering solutions. As we explore the intricate details of plant-based decellularization, we gain a deeper understanding of its potential, challenges, and transformative implications for the future of regenerative medicine.

**Fig. 2:**
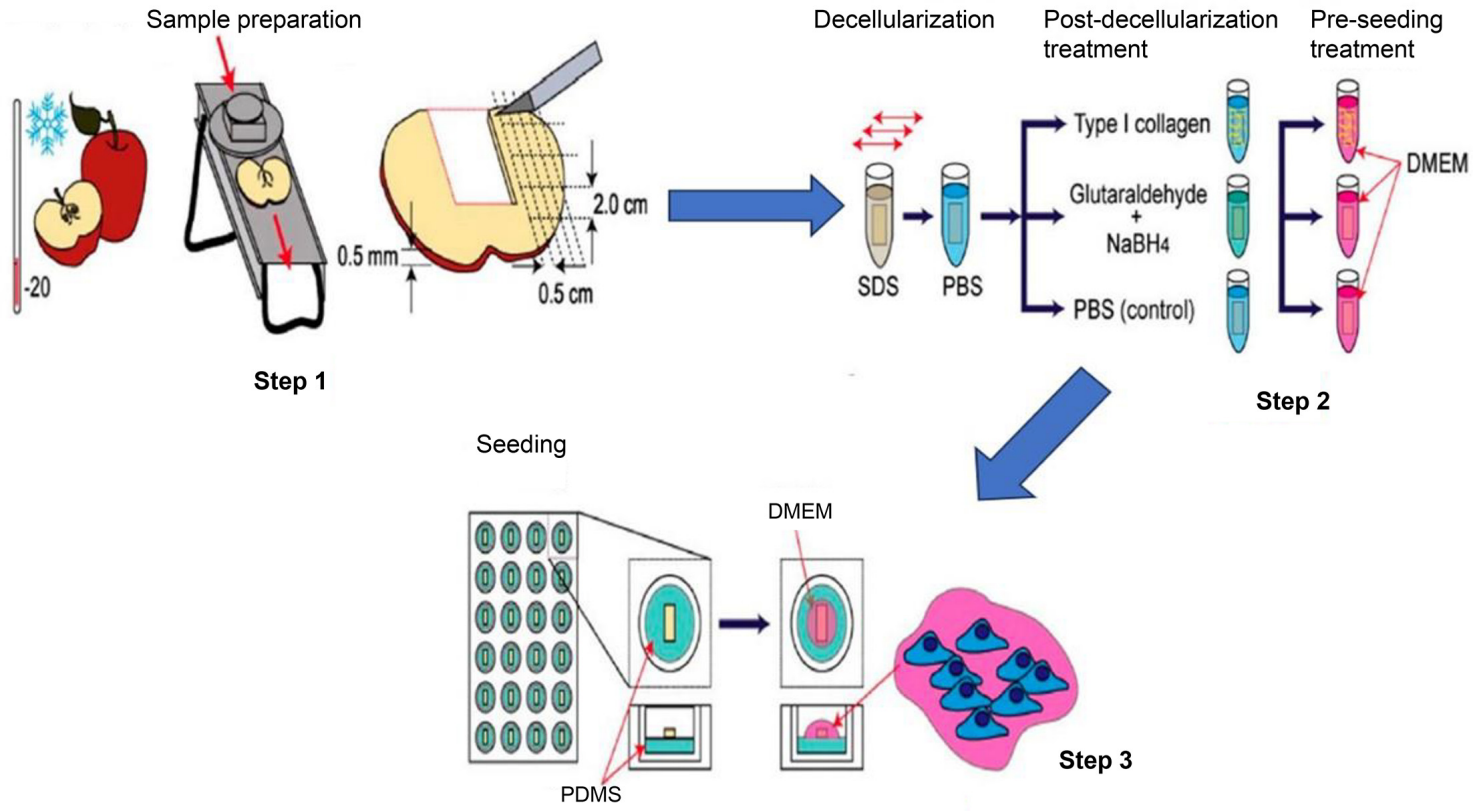
Apple-derived cellulose scaffold generation for 3D mammalian cell culture. McIntosh red apples were subjected to a controlled drying process at 220°C for up to 5 min to rigidify the outer hypanthium tissue. Following this, the apples underwent precise slicing using a mandolin slicer, ensuring thin and uniform sections while removing the cores. Subsequently, uniform segments measuring 2.0 × 0.5 cm were excised from the sliced apples and individually placed in microcentrifuge tubes for further processing. Decellularization of these apple segments ensued, involving the removal of cellular components while preserving the structural integrity of the cellulose matrix. Post-decellularization, the segments underwent surface modification with various chemistries. These modifications included coating with Type 1 collagen, chemical cross-linking using glutaraldehyde, or incubation in phosphate-buffered saline (PBS).The treated segments, now transformed into scaffolds, were introduced into mammalian cell culture medium (DMEM) and incubated for 12 h under standard tissue culture conditions (37°C, 5% CO_2_). This incubation period allowed for equilibration and preparation of the scaffolds for subsequent cell seeding. For cell seeding, polydimethylsiloxane (PDMS)-coated 24-well plates were utilized, with each well containing 40 ml of cell suspension. The seeded scaffolds were incubated for 6 h to facilitate cell attachment and initial colonization. Subsequently, the wells were filled with DMEM and maintained for up to 12 weeks, providing an extended culture period to assess cell growth, viability, and functionality within the scaffolds. (Reproduced, with permission, copyright 2014, PLOS).

Modulevsky *et al*. (2014) used sodium dodecyl sulfate (SDS) detergent for preserving cellulose extracellular matrix (ECM) integrity in apple tissue as the initial steps in plant-based decellularization (Fig. 2). Subsequent studies introduced variations, with Gershlak *et al*. (2017) supplementing the SDS wash, Hickey *et al*. (2018) incorporating a CaCl_2_ treatment, and Adamski *et al*. (2018) comparing SDS-based decellularization to a detergent-free method. Innovative approaches, such as Varhama *et al*.’s (2019) boiling and brushing method and Thippan *et al*.’s (2019) inclusion of a freeze-thaw cycle, demonstrated adaptability to different tissues. Additionally, DNAase 1 was used to decellularize tobacco BY-2 cells and rice cells, showcasing the versatility of plant-based decellularization. These diverse protocols highlight nuanced strategies developed to address specific challenges in plant tissue decellularization, collectively contributing to the evolution of this transformative field.

### Crucial Aspects of Scaffold Fabrication

Fabrication of scaffolds is pivotal in tissue engineering and influences the success of regenerative medicine applications. This article provides an in-depth exploration of the essential aspects of scaffold fabrication, illuminating key factors that influence their design, functionality, and overall effectiveness in tissue engineering.

**(a) Material Selection**

The choice of material for scaffold fabrication is paramount and must be aligned with the specific tissue or organ application [30]. Critical factors such as biocompatibility, biodegradability, and mechanical properties significantly influence this selection process.

**(b) Structural Design**

Structural design, including aspects such as pore size, interconnectivity, and overall architecture, substantially influences cell behavior and tissue regeneration. Emerging research, including the work, explores advanced structural designs that mimic the native ECM, fostering improved cell adhesion, proliferation, and differentiation [31].

**(c) Manufacturing Techniques**

Various manufacturing techniques, such as electrospinning, 3D printing and freeze-drying, are employed in scaffold fabrication. Comparative analyses of these techniques offer insights into their respective strengths and limitations, aiding researchers in selecting the most suitable method for their specific needs [32].

**(d) Surface Modification**

Surface modification of scaffolds can enhance bioactivity and cell–scaffold interactions [33]. Strategies such as biomolecule coating or bioactive nanoparticle incorporation are explored, highlighting the importance of surface modification in scaffold tailoring for specific tissue environments.

**(e) Biological Functionalization**

Incorporating biological cues into scaffolds is crucial for tissue regeneration. The role of biological functionalization in scaffold fabrication, emphasizes the integration of growth factors, peptides, and other signaling molecules [34].

**(f) Quality Control and Standardization**

Ensuring the reproducibility and consistency of scaffold fabrication is crucial for clinical translation. The importance of quality control measures and standardization protocols for scaffold fabrication, addressing challenges, and proposing guidelines for maintaining manufacturing reproducibility is critical [35].

In addition, the production of scaffolds for tissue engineering presents a number of challenges and considerations that must be carefully addressed to achieve successful outcomes. These challenges summarized below emphasize the intricacies of scaffold preparation and their impact on various aspects of tissue engineering.

**(i) Scaffolding Methods, Tissue Types, and Applications**

Scaffold preparation raises critical issues related to pore size, focusing on specific cell types and engineered tissues. The choice of scaffolding methods is closely linked to the intended tissue application and requires scalability for clinical and commercial use. The development of scalable manufacturing processes in accordance with good manufacturing practice (GMP) standards is crucial for the clinical application of tissue engineering approaches [15]. These considerations extend to product delivery, storage and off-the-shelf availability, taking into account clinicians’ preferences for ease of use.

**(ii) Biodegradability and Biocompatibility**

Biodegradability and biocompatibility are essential properties for cell adhesion and propagation. Cytocompatibility testing, in vivo formulation evaluation and analyses of bioactivity and biocompatibility are essential. Scaffolds should induce a minimal immune response after grafting in order to avoid an inflammatory response that may hinder healing [36]. The choice of materials, including plant-based sources, has been explored for their favorable properties, such as high biocompatibility and bioactivity, making them suitable for tissue engineering.

**(iii) Mechanical Property**

Scaffold mechanical properties must align with the anatomical site of implantation, necessitating strength for surgical handling during grafting [23]. Tailoring mechanical integrity is a significant challenge, particularly in cardiovascular and orthopedic applications where long-term mechanical support is crucial. To ensure both cellular infiltration and vascularization, achieving a balance between mechanical characteristics and porous architecture is critical [37].

**(iv) Architecture**

The structural architecture of scaffolds requires an interconnected pore structure and high porosity [27]. This facilitates cellular penetration, nutrient dispersal, and waste product diffusion. The size of the pores must strike a delicate balance to allow cell migration while maintaining specificity for appropriate ligand binding [38]. A porous interconnected structure is necessary for the elimination of waste products without hindering the surrounding tissues.

**(v) Clinical Status Evaluation**

Clinical evaluation is essential to validate scaffold efficacy [10]. Micro-computed tomography imaging and histological analyses are performed to assess organ defect repair. Challenges include limited sample sizes, lack of randomized control groups, and long-term study limitations [21]. Overcoming these limitations is necessary to detect rare adverse events and assess the long-term benefits of interventions.

## Modification of Decellularized Plant Tissues

Modification of decellularized plant tissues is at the forefront of advances in tissue engineering, reflecting a transformative strategy in dynamic regenerative medicine. Decellularization, the removal of cellular components from biological tissues, has gained prominence due to its potential to create scaffolds suitable for regenerative purposes [39]. This paradigm shift focuses on harnessing the distinctive attributes of plant tissues, an abundant and sustainable source, to form the basis for engineered structures. The title “Modification of Decellularized Plant Tissues” encapsulates the pursuit of innovative strategies to enhance and tailor these plant-derived scaffolds [40]. In tissue engineering, researchers investigate the complex process of modifying decellularized plant tissues to address specific challenges and optimize their applicability. The term “modification” encompasses a spectrum of interventions, ranging from surface alterations to structural enhancements, all aimed at tailoring plant-derived scaffolds to meet diverse tissue and organ requirements [41]. This interdisciplinary work combines expertise in biology, materials science, and engineering to overcome the complexities of plant tissue modification. Researchers have explored a myriad of possibilities, including the incorporation of bioactive molecules, structural adjustments, and surface modification to refine the properties of decellularized plant tissues [42]. The overarching goal is to create scaffolds that not only mimic the natural ECM but also exhibit enhanced functionalities for promoting cell adhesion, proliferation, and differentiation [28]. Furthermore, this modification process aims to address inherent limitations of plant-derived materials, such as mechanical strength and degradation kinetics, making them more compatible with the dynamic demands of tissue engineering [43].

Exploring the modification of decellularized plant tissues holds promise for advancing regenerative medicine, aligning with sustainability principles by offering an eco-friendly alternative to traditional scaffold materials [44]. This scientific journey, encapsulated by the title, highlights the modification of decellularized plant tissues as a transformative strategy, opening new avenues for creating advanced scaffolds with profound implications for the future of tissue engineering [45]. The comprehensive exploration of plant tissue modifications not only compensates for the absence of native ECM proteins but also underscores the adaptability and versatility of cellulose-based biomaterials in biomedical applications [46]. The integration of ECM-mimicking modifications extends the potential applications of decellularized plant tissues, rendering them more suitable for meeting the complex demands of tissue engineering and regenerative medicine.

## Emerging Applications of Decellularized Plant-Based Scaffolds

The use of porous 3D scaffolds to provide a suitable environment for the generation of tissues and organs is vital for tissue engineering applications and for the exploration of novel cellular models for biomedical research [43]. For these purposes, several biomaterials have been explored, including ceramics, metals, bioactive glasses, animal-derived tissues, and polymers. Only recently, however, have plants and plant-based polymers emerged as relevant biomaterials [47].

### Tissue Engineering

**Vascularization of Decellularized Plant-Based Scaffolds**

**Fig. 3:**
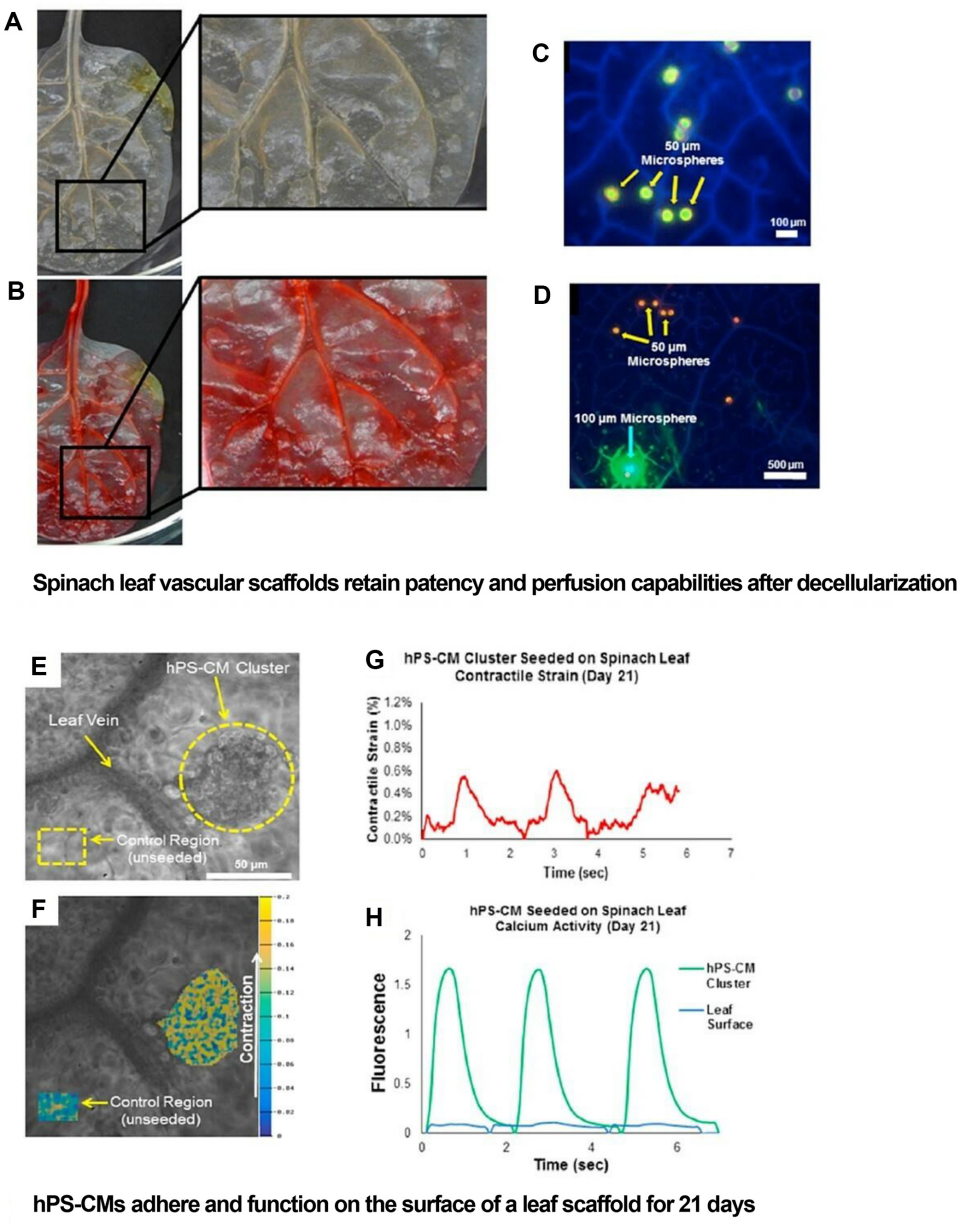
Spinach Leaf Vascular Scaffolds in 3D Mammalian Cell Culture. Spinach leaf vascular scaffolds demonstrate remarkable retention of patency and perfusion capabilities post-decellularization, enabling successful adhesion and function of human pluripotent stem cell-derived cardiomyocytes (hPS-CMs) for 21 days. (**A, B**) Display the decellularized leaf before and after Ponceau Red perfusion, highlighting the preservation of the vascular architecture crucial for cellular perfusion and viability. (**C, D**) Fluorescence images depict leaf vasculature perfused with beads, illustrating the retention of 50 and 100 μm spheres within the vascular network. Scale bars indicate dimensions, crucial for assessing microvascular architecture: (**C**) 100 μm, (**D**) 500 μm. (**E**) hPS-CMs adhere to the leaf scaffold surface, forming distinct cell clusters critical for tissue organization and function. The scale bar denotes 50 μm, enabling precise assessment of cellular arrangements. (**F**) Contractile strain, indicative of cellular functionality, is visualized through a heatmap, providing insights into the dynamic behavior of hPSCMs within the scaffold environment. (**G**) Day 21 strain values reveal a diminished contractile strain magnitude, suggesting potential cellular maturation and adaptation within the scaffold over time. (**H**) Relative changes in fluorescent signals relative to the leaf surface are visualized, offering quantitative data on cellular distribution and viability throughout the scaffold. This comprehensive analysis demonstrates the efficacy of spinach leaf vascular scaffolds in supporting 3D mammalian cell culture, showcasing their potential in tissue engineering and regenerative medicine applications. The ability to retain vascular patency and perfusion, coupled with successful hPS-CM adhesion and function, underscores the utility of plant-derived scaffolds as biocompatible substrates for advanced cell culture studies. (Reproduced, with permission, copyright 2017, Elsevier)

Gershlak *et al*. (2017) employed a novel strategy, utilizing leaf vasculature as a perfusion platform for larger graft bioengineering [21]. This approach involved creating a prevascularized scaffold for tissue engineering applications by decellularizing plant tissue. Different plant species required distinct modifications to perfusion-based decellularization, resulting in varying scaffolding geometries [48]. The plant scaffolds retained their patency and capability to transfer microparticles following decellularization. In this study, human endothelial cells were used to recellularize plant scaffolds, colonizing the inner surfaces of the plant vasculature [29]. Cardiomyocytes, derived from human pluripotent stem cells and human mesenchymal stem cells, adhered to the external surfaces of plant scaffolds. Over a 21-day period, these cardiomyocytes displayed contractile activity (Fig. 3) and calcium handling ability. The primary objective was to determine if the decellularization process preserved the integrity and patency of the leaf vasculature. Decellularized spinach leaves were perfused with Ponceau Red through a cannula. The entire leaf vasculature was suffused with Ponceau Red, though minor leakage was observed (Fig. 3A and B). Furthermore, the perfusate penetrated and traversed the leaf vein’s smaller branches, indicating that the leaf ’s microvasculature was largely unaffected. Similar observations were made when examining beads entrapped within the plant vascular networks (Fig. 3C and D); specifically, only microspheres with diameters of 50 and 100 μm were found trapped within the plant tissue. Collectively, these results suggest that the leaf vasculature allows passage of particles comparable in size to red blood cells. Cardiomyocytes derived from human pluripotent stem cells (hPS-CMs) showed promising results in terms of adhesion and functional activity on spinach leaf surfaces. On Day 21, the intensity of fluorescent signals in hPS-CM clusters was monitored over several contractile cycles and compared with the signal from the leaf ’s surface (Fig. 3E-H). The calcium signal emanating from the hPS-CM cluster was more intense than that from the leaf ’s surface, confirming that the hPS-CMs were not an autofluorescence artifact and maintained their calcium handling capability when seeded onto the leaf scaffolds. The supporting structure of the cellulose architecture, along with the conveniently sized xylem and phloem channel diameters of the spinach leaf, could contribute to the creation of a prevascularized scaffold. This scaffold has the potential to be biocompatible, cost-effective, and sustainable for scalable tissue applications, such as transplantation and regenerative medicine. Robbins *et al*.(2020) modified the surface biochemistry of the scaffold preparation process, originally developed by Gershlak *et al*. (2017), by investigating the necessity of an ECM coating, such as collagen IV or fibronectin, for developing a contractile, decellularized, hiPS-CM infused spinach leaf scaffold intended for cardiac tissue regeneration or grafting. The scaffolds loaded with hiPS-CMs exhibited no significant alteration in attachment, contractility, or sarcomeric length due to the absence of a coating. Furthermore, the necessity of fetal bovine serum (FBS) was evaluated, and a method to eliminate FBS from the hiPS-CM seeding medium was tested. The removal of FBS did not affect contractility, yielding results comparable to those of the original protocol: formation of spherical cell clusters and elongated sarcomeric bands. The in vitro concept involved arranging decellularized leaves or stems adjacent to each other in a gel, aiming to induce the sprouting of blood vessels between them in response to chemoattractants or growth factors [49]. The pro-angiogenic factors were anticipated to be sourced from human umbilical vein endothelial cells (HUVECs), building on successful experiments with hMSCs [50]. Various configurations and mediums were explored to develop a gel-scaffold complex that would support proper cell attachment, viability, and the formation of nascent vessels, emulating the initial stages of angiogenesis [51]. Although these configurations were not experimentally validated, the study confirmed cell viability and adhesion to a decellularized spinach leaf scaffold, using red fluorescent imaging to visualize quantum-dot loaded mesenchymal cells [52]. The scaffold’s porosity was evaluated by pumping microspheres through the spinach stem and quantifying the leaked microspheres. Fibrin gel was identified as an optimal choice due to its properties conducive to wound healing [30]. The proposed design holds promise for application in large-scale organ engineering.

**Mimicking Skeletal Tissue with Decellularized Plants**

**Fig. 4:**
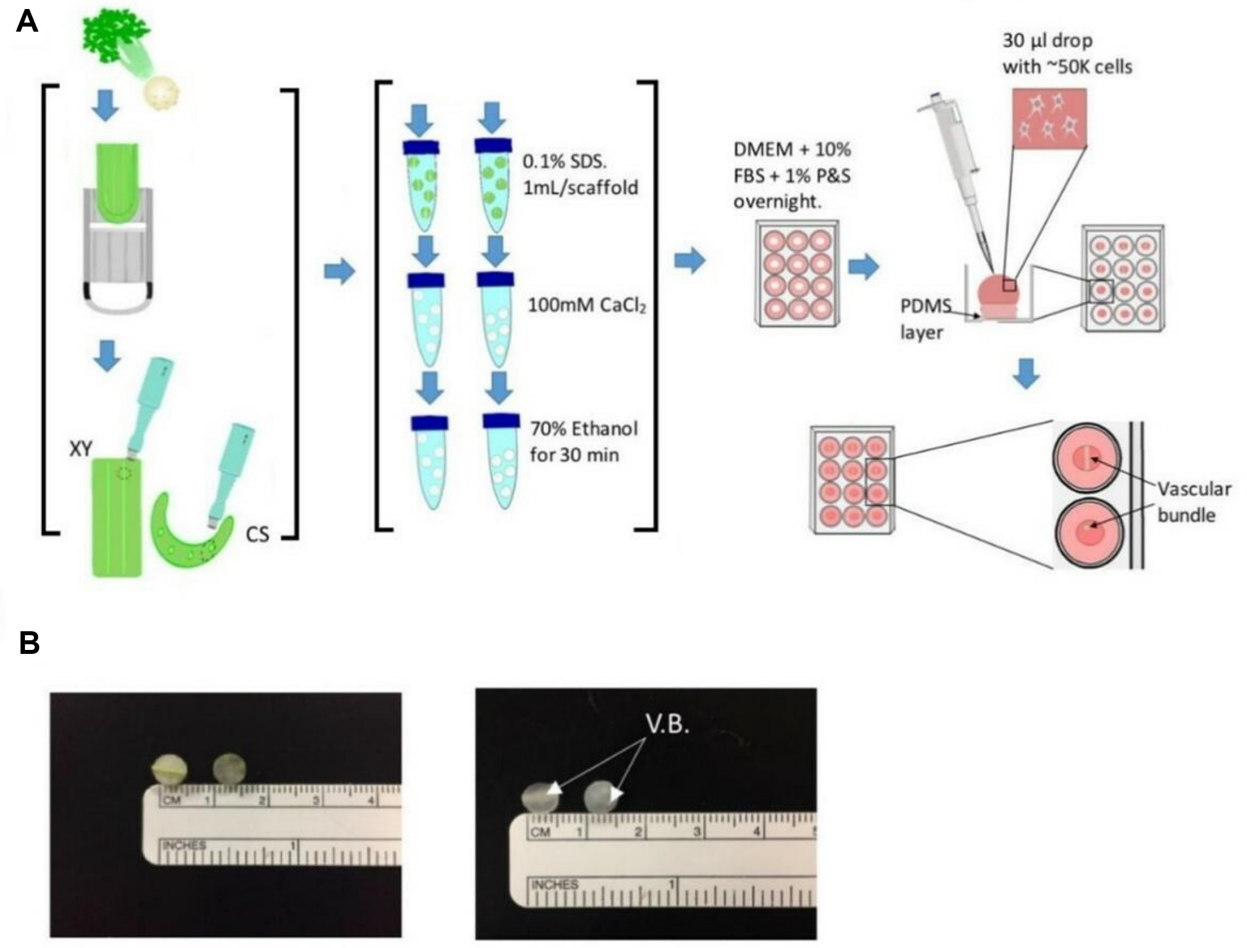
Celery (*A. graveolens*) Scaffold Preparation and Cell Seeding. (**A**) This visual representation depicts the process of celery scaffold preparation, crucial for subsequent tissue engineering applications. (**B**) The samples obtained were 6mm wide with a thickness of 2.15 ± 0.15 mm. "XY" denotes scaffolds cut longitudinally (left) relative to the celery stalk, while "CS" corresponds to cross sections (right). After a 3-day incubation period in 0.1% SDS, the samples became clear, indicating successful decellularization. (**C**) Decellularized scaffolds served as substrates for cell seeding, with approximately 50,000 cells applied and left on the scaffold for 4.5 h. This crucial step initiates cell attachment and colonization onto the scaffold matrix. The presence of the vascular bundle (V.B.) within the scaffold underscores its anatomical relevance and potential for vascularized tissue engineering approaches. This method offers a promising platform for developing tissue-engineered constructs using natural plant-based scaffolds. The transparency achieved post-decellularization facilitates cell visualization and analysis, aiding in the evaluation of cell behavior and tissue formation. This comprehensive approach bridges the gap between plant biology and tissue engineering, offering novel strategies for scaffold fabrication and cell seeding techniques. Further exploration of celery-derived scaffolds holds significant promise for regenerative medicine applications and organ-ona- chip technologies. (Reproduced, with permission, copyright 2020, bioRxiv)

In their 2020 study, Campuzano *et al*. utilized the natural architecture of decellularized celery (Apium graveolens) as a cellulose-based substrate scaffold to confine or guide precursor C2C12 murine myoblasts into uniaxial orientation in the presence of differentiation media [44]. They prepared the *A. graveolens* stalk by cutting it cross-sectionally and longitudinally to create the scaffolds (Fig. 4A). The morphology of the cross sections appeared amorphous, while the longitudinal scaffolds exhibited well-aligned vascular bundle grooves. The scaffolds lost their green color after three days in 0.1% SDS, indicative of the removal of biological components (Fig. 4B). To facilitate the elimination of SDS, scaffolds were treated for a full day with a 100 mM concentration of CaCl_2_. Successful cell-matrix attachment was achieved using adhesion proteins found in FBS without the need for additional biofunctionalization. The xylem and phloem channels in the decellularized celery, ranging from 10 to 100 μm in diameter, provided an appropriate platform for seeding cells in an orientation that promoted even distribution along with the vascular bundle. After ten days in proliferation media, F-actin filaments were observed aligning along the longitudinal axis of the vascular bundle. However, their confluence was deemed insufficient for fusion and myotube formation, despite the fact that myoblast alignment facilitated the expression of troponin T, myogenin, and myosin heavy chain II, all crucial for sarcomeric unit formation [20]. Two significant limitations of the cellulose-based platform were identified: the absence of biochemical cues typically found in the ECM and the mechanical stiffness of decellularized plant tissue compared to mammalian ECM [25]. Nevertheless, this cellulose-based scaffold represents a biocompatible and minimally immunogenic alternative for 3D tissue engineering in vitro and stem cell differentiation research. Its structural resemblance to the ideal configuration of myoblasts positions it as a promising avenue for studies on in vitro muscle myogenesis [53].

**Bone Tissue Engineering with Decellularized Plant-Based Scaffolds**

**Fig. 5:**
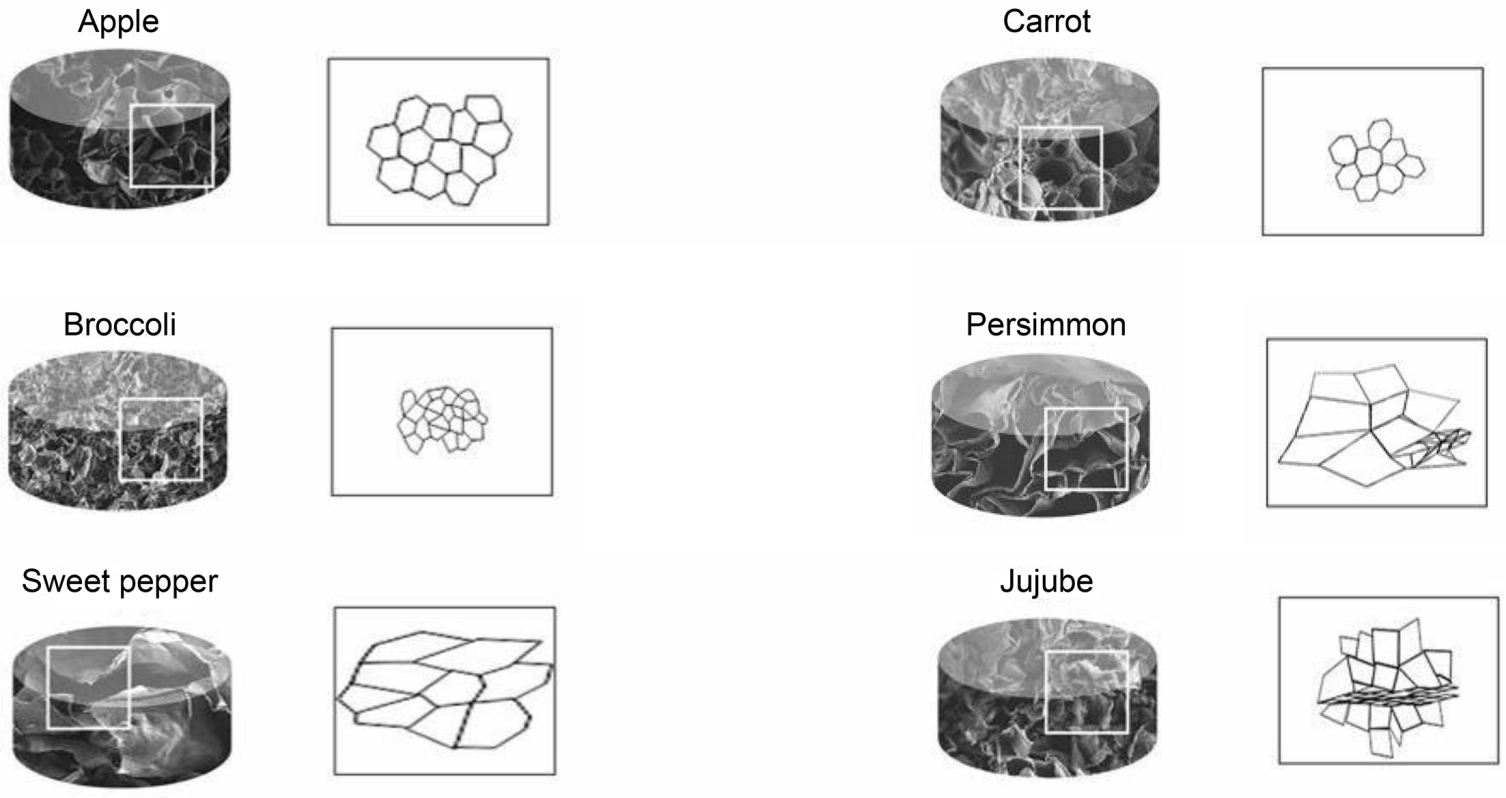
Induced pluripotent stem cells (iPSCs) cultivated in apple-derived scaffolds. The diagram showcases a range of plant scaffolds, each characterized by distinct shapes and pore sizes. These attributes are critical for fostering the growth and development of iPSCs within the scaffolds. Understanding the morphology and porosity of these scaffolds is essential for optimizing cellular interactions, which is particularly important in tissue engineering and regenerative medicine applications. By utilizing apple-derived scaffolds, researchers aim to leverage the unique properties of plant-based materials to support iPSC growth and differentiation in biomedical contexts. This visualization provides valuable insights into how scaffold architecture influences cell behavior and organization, offering potential avenues for enhancing the efficacy of iPSC-based therapies and tissue engineering strategies. (Reproduced, with permission, copyright 2019, Scientific Reports, Springer)

Lee *et al*. (2019) created a bone-like tissue by cultivating pluripotent stem cells (hiPSCs) stimulated to differentiate into osteoblasts on decellularized scaffolds derived from various plants [54]. The effectiveness of this approach was gauged by evaluating the expression of relevant cellular markers and the extent of calcium-specific staining. Studies have considered a structural framework for bone tissue cultivation, based on plant scaffolding. In their investigation, Lee *et al*. evaluated the suitability of decellularized apples, carrots, persimmons, and other fruits with pore sizes of approximately 300 μm for supporting the 3D growth of induced pluripotent stem cells (iPSCs) into osteoblasts (Fig. 5). The apple scaffolding, characterized by regular 300 μm pores, demonstrated the most favorable construct development among the plant scaffolds tested. iPSCs can be derived from any source of precursor cells by reversing the differentiation process using Yamanaka factors. This method facilitates the generation of organoids and offers a potential approach for extracting bone-like tissue [50]. The hiPSCs exhibited varying degrees of distribution, development, and survival across the different scaffolds, with the apple scaffold showing effective adherence and spread [55]. The iPSCs in the apple scaffold were incubated in a solution of FBS, low glucose DMEM with ascorbic acid, dexamethasone, and β-glycerol phosphate, subsequently placed in PDMS-coated wells, and cultivated with osteogenic differentiation media [54]. The resultant tissue resembled bone and showed potential for transplantation in a rat calvarial deficiency model, aiding in the creation of calcified tissue. This strategy indicates a feasible method for efficiently producing mineralized bone, contingent on the regularity and size of the scaffold pores.

Latour *et al*. (2020) explored bone tissue engineering using decellularized apple hypanthium tissue. The pore size of the tissue (100–200 μm) and its physical characteristics were akin to those of trabecular bone [39]. The study assessed the mineralization capability and mechanical properties of the apple-derived platform, coated with mammalian cells, against a bare surface and a surface coated with collagen I (composite hydrogel scaffold) [39]. High-speed resonant confocal laser scanning microscope imaging and histologic analysis revealed that preosteoblastic cells adhered to and distributed uniformly around the pores of both apple and composite hydrogel scaffolds. SEM imaging showed calcium aggregation around the pore edges. The expression of alkaline phosphatase (ALP) was evaluated using BCIP/NBT and Alizarin Red S staining, which indicated deeper staining in both platforms post-differentiation [39]. Despite the collagen coating, the increase in ALP and calcium deposits suggested successful osteogenesis. Following the culture of MC3T3-E1 cells, Young’s modulus of both scaffolds also increased, which is indicative of mineralization due to osteoblast differentiation. Given that Young’s moduli of trabecular and cortical bones are higher than those observed in the scaffolds, this method is primarily suitable for non-weight-bearing bones, such as those in the hands and wrists. The apple-derived matrix allows precursor osteoblast cells to invade, proliferate, and differentiate in and around the pores, which is beneficial for the functional and autologous engineering of bone tissue [37].

### Regenerative Medicine

Regenerative decades have seen a growing interest within scholarly communities in the use of plant proteins as biodegradable and cytocompatible substances [56]. These proteins can be synthesized into fibers, hydrogels, micro- and nanoparticles, porous structures, and composites, exhibiting qualities suitable for tissue regeneration [43]. Notably, traditional tissue engineering materials often involve toxic solvents and non-environmentally friendly polymers, positioning regenerative medicine among the last fields to embrace green or eco-friendly procedures [57]. Plant protein scaffolds, like zein, soy protein, and wheat gluten, offer a sustainable alternative for regenerative medicine applications thanks to their mechanical properties, biocompatibility, and water stability [58]. The utilization of safe, eco-friendly materials and methods reduces the toxic and hazardous impacts of scientific research while minimizing energy consumption and waste production from various synthetic processes. One study demonstrated the production of a cross-linked porous scaffold based on a chitosan-soy protein blend system, employing the sol-gel method combined with freeze-drying [36]. The use of tetraethyl orthosilicate (TEOS) as a cross-linker enhanced the mechanical stability, degradation rate, and surface energy of the scaffolds. The silanol groups, associated with bone tissue engineering applications, may promote a mineral-type apatite surface under physiological conditions [52]. These scaffolds have also been investigated for cartilage tissue engineering applications. Another study developed porous membranes based on a cellulose-soy protein isolates (SPI) blend technique [22]. This approach involved blending SPI with hydroxyethyl cellulose (HEC), a water-soluble cellulose variant, to improve in vivo biodegradability of the composite scaffolds [59]. Epichlorohydrin (ECH) was utilized to cross-link SPI and HEC. The resulting films were biodegradable in vivo, with the rate of decomposition adjustable via the SPI concentration. Films containing over 30% SPI exhibited enhanced mechanical properties, water resistance, and appropriate biodegradability [60]. Gliadin and glutenin, two proteins from wheat gluten, were used to create membranes for potential tissue engineering scaffolds [61]. These films exhibited varied behaviors in water degradation and their ability to support fibroblast adhesion and proliferation. Submersion in water at pH 7.4 and 37°C for 15 days led to weight losses of 90% and 50% for wheat glutenin and gliadin, respectively [61]. Fibroblast cells found gliadin cytotoxic, whereas glutenin, devoid of gliadin and starch, was cytocompatible and significantly enhanced fibroblast cell adhesion and proliferation.

Particle leaching is a popular method for achieving regulated porosity in zein scaffolds [62]. In a study, a salt-leaching technique was employed to transform zein into a porous scaffold for potential bone applications. After 14 days of in vitro incubation, the zein scaffolds exhibited excellent porosity (75.3%–79.0%), strong pore interconnectivity, high mechanical properties, and a degradation rate of 89% with pepsin and 36% with collagenase [63]. The scaffolds facilitated the adhesion, growth, proliferation, and differentiation of hMSCs [64]. Another study used a solvent casting/particulate leaching approach to create a zein porous scaffold suitable for the growth of periodontal ligament cells (PDLCs), achieving appropriate porosity (64.1%–78.0%) and biocompatibility [43]. Additionally, a study utilized a similar method to construct porous scaffolds from zein/Polycaprolactone (PCL) composites for bone regeneration [62]. These biocomposite scaffolds displayed a well-connected network and a high porosity of around 70%. Compared to PCL scaffolds, zein/PCL scaffolds were more hydrophobic and degraded more rapidly after 28 days in PBS [65]. This research indicated that the degradation rate of the scaffolds could be tailored to match tissue regeneration rates by adjusting the zein concentration in the composite.

### Cosmetics and Skin Care

Bioactive peptides (BP), specific protein fragments, play pivotal roles in the physiological functions of most living organisms. These BPs are involved in cellular signaling, developmental regulation, and defensive responses in plants. A study conducted on H_2_O_2_-stressed dermal human fibroblasts utilized an Avena sativa (oat) peptide-rich preparation derived from oat bran through enzymatic hydrolysis. The findings revealed that this preparation effectively reduced oxidative stress-induced cell damage by enhancing the activity of the enzyme Superoxide dismutase and inhibiting malondialdehyde (MDA) levels. Furthermore, *Triticum vulgare* (wheat) protein hydrolysates have been produced and suggested for use as conditioning agents in skin and hair care products [66]. Another instance is a *Solanum tuberosum* (potato) protein hydrolysate, which, through a hydrolytic process, stimulated response mechanisms in skin cells. When added to the culture medium, this potato hydrolysate increased long-chain fatty acid levels and accelerated lipid metabolism in skin keratinocytes [67].

## Future Perspectives

The promising outcomes of plant-based decellularization necessitate further research and development to fully harness its potential in regenerative medicine. Future studies should focus on refining decellularization techniques, thoroughly understanding the long-term effects of plant-based scaffolds, and scaling the methodology for clinical use. Comprehensive investigations into modifying decellularized plant tissues are essential to enhance their mechanical properties and biocompatibility. Creating a summary table of relevant publications detailing plant species, decellularization procedures, and key findings would be invaluable for researchers and practitioners in the field. Such a resource would consolidate knowledge, foster collaboration, and guide further exploration into diverse applications of plant-based decellularization. The advancement of plant decellularization should continue exploring various plant species and tissues to expand the range of potential scaffold materials. Active research in innovative scaffold fabrication techniques, including emerging applications in perfusable structures, is also crucial. Bridging the gap between biological sciences and engineering is key to establishing plant-based decellularization as a standard in tissue engineering. Ultimately, plant-based decellularization represents a revolutionary method with the potential to significantly impact regenerative medicine. By creating sustainable, cost-effective, and perfusable tissue engineering structures, this approach could revolutionize the field and contribute to next-generation therapeutics.

## Conclusion

Plant-based decellularization represents a transformative and eco-friendly advancement in tissue engineering, offering innovative solutions for scaffold design and vascularization challenges. This article comprehensively examined the multifaceted aspects of plant-based decellularization, underscoring its potential to produce sustainable, cost-effective, and perfusable architectures for tissue engineering. The utilization of plant-derived materials, noted for their abundance and rapid growth, addresses critical concerns regarding sustainability and green chemistry, positioning this technology as a significant breakthrough in the field of regenerative medicine. The investigation covered essential components, including the methodologies for plant decellularization, challenges in scaffold production, various modification approaches, and case studies that demonstrate the practical applicability of this technology in tissue engineering. The article delved into both the advantages and challenges associated with plant-derived polymers, highlighting the significant potential of natural biopolymers to influence cell behavior and enhance biocompatibility. It discussed strategies such as post-spinning cross-linking and the blending of natural with synthetic polymers as methods to counteract inherent limitations, such as poor mechanical properties and rapid biodegradability. A unique and pivotal advantage of plant-based decellularization is its capacity to exploit the similarities between plant and animal vascular networks. By emulating the intricate, tapered, and branching network design akin to the human cardiovascular system, plant-based decellularization presents an innovative and groundbreaking strategy for creating perfusable scaffolds. This approach effectively addresses a critical limitation in contemporary bioengineering methods. The potential to develop scaffolds that support perfusion not only advances the field of tissue engineering but also paves the way for translating these solutions into clinical applications.

## Figures and Tables

**Table 1:** Type, Decellularization Method, Modification, Relevant Feature, and Potential Application of Each Scaffold Experiment.

Plant Species	Decellularization Method	Modification	Relevant Feature	Potential Application	Reference
Apple (McIntosh red Plant)	0.5% SDS for 12 h	Type 1 Collagen coating	High Porosity 3D scaffold	Mammalian Cell Culture	Modulevsky *et al*. 2014
Leek (*Allium porrum*)	1% SDS for 5 days	Graphene oxide coating	Morphological structure	3D Scaffold	Toker *et al*. 2018
Spinach	10% SDS for 5 days, 1% Triton X-100 for 1 day, 0.1% Triton X-100 for 1 day, and 10% sodium hypochlorite solution for 4 h	Gelatin coating	Vasculature	Angiogenesis	Dikici *et al*. 2019
Aptenia cordifolia Schwant leaves	10% SDS for 24 h followed by 0.5% Triton X-100 containing 1.2% sodium chlorite bleach for 24 h (perfusion)	Grafting	Vasculature	Vascularization	Wang *et al*. 2020
Apple (McIntosh Red apple)	0.1% SDS for 48 h	1. Temporary molding with gelatin: 2 Permanent molding with collagen	High Porosity; Versatility	Template for blood vessel formation (in vivo)	Hickey *et al*. 2018
Spinach and Parsley; Artemisia annua leaves and Peanut hairy roots	a. 10% SDS for 5 days followed by 0.1% Triton-X-100 in a 10% sodium chlorite bleach for 48 h; b. Perfusion; b. Soaking	Fibronectin coating	Vasculature	Cardiac Tissue Engineering	Gershlak *et al*. 2017
Spinach	1% SDS for 24 h followed by 0.1% Triton X-100/10% bleach for 24 h (perfusion)	1 - Fibronectin coating; 2. Collagen IV coating	Vasculature	Cardiac Tissue Engineering	Robbins *et al*. 2020
Apple (*Malus domestica*), Carrot (*Daucus carota*), Celery (*Apium graveolens*)	0.1% SDS for 48 h	Poly (L-lysine)	Porosity and mechanical properties	Adipose tissue (apple), Bone tissue engineering (carrot), Tendon (celery)	Contessi Negrini *et al*. 2020
Apple (McIntosh Red apple)	0.1% SDS for 48 h	NA	High Porosity 3D scaffold (in vivo)	Subcutaneously Implanted Plant-Derived Cellulose Biomaterials	Modulevsky *et al*. 2016
Apple, Broccoli, Sweet pepper, Carrot, Persimmon, Jujube	0.5% SDS for 48 h	NA	Size and shape of pores	Osteogenesis (in vivo)	Lee *et al*. 2019
Apple (McIntosh Red apple)	0.1% SDS for 48 h	Collagen coating	High Porosity	Bone Tissue Engineering	Latour *et al*. 2020
*Alathea zebrina*, *Anthurium waroquaenum*, *A. magnificum*, *Laelia ancepts*, Bamboo, Parsley, Schoenoplectus tabernaemontani	10% SDS for 5 days followed by 0.1% Triton-X-100 in a 10% bleach for 48 h	1 - Functionalized by RGDOPA; 2 - Mineralization	Hierarchical, hydrophilic, and interconnected ultrastructure	3D Scaffold	Fontana *et al*. 2017
Celery (*Apium graveolens*)	0.1% SDS for 72 h	NA	Vascular Bundle Channels	Skeletal muscle	Campuzano *et al*. 2020
Piper betel, *Sauropus androgynus*, *Basella alba*, *Azadirachta indica*, *Centella asiatica*, *Mentha spicata* Leaves |	0.1% Triton or 0.1% EDTA for 5 days	NA	Vasculature	Vascularization	Thippan *et al*. 2019
Tobacco Bright Yellow-2 (BY-2) cells, Rice cells (Oryza sativa L.), Tobacco hairy roots (*N. Tabacum*)	DNase in combination with lyophilization	NA	Ease of genetic modification	Pharmaceuticals	Phan *et al*. 2020
